# Time-dependent power laws in the oxidation and corrosion of metals and alloys

**DOI:** 10.1038/s41598-022-10748-1

**Published:** 2022-04-28

**Authors:** Makoto Itoh

**Affiliations:** Unaffiliated, 11-995 Yamanokami, Yurihonjo, Akita 015-0014 Japan

**Keywords:** Engineering, Materials science, Physics

## Abstract

Using the equations which describe the oxide thickness as a function of the oxidation time and temperature in the thermal oxidation of Si, various experimental results on the oxidation and corrosion of metals and alloys available in the literature are analyzed. By the analyses, it is found that the weight loss of copper by atmospheric corrosion and the weight gains of austenitic stainless steel and Ni–Cr alloy by high temperature oxidation follow a time-dependent power law in which both diffusion and reaction are involved. It is also found that the pitting corrosion of aluminum alloys by the immersion with seawater and the high-temperature oxidation of Al(431) follow a time-dependent power law of a reaction-limited kind. In addition, an estimation is given of the activation energy for the pitting corrosion of mild steel by the immersion with seawater.

## Introduction

The mechanism on the corrosion of metals and alloys has often been investigated by the electrochemical analyses^1–3^. Although such analyses are certainly useful in studying short-term phenomena, investigations of long-term behaviors sometimes become necessary when one studies corrosion or oxidation of metals at high temperatures^4^ or in the environmental condition of marine atmosphere^2^. This is particularly true if one studies the heat-resistance properties of stainless steel used for constructing a blast furnace or a nuclear reactor. For such studies, analyses of experiments based on the theories of electrochemistry alone are not sufficient, and a physical approach should be taken. This is easily recognized by the fact that the use of the parabolic law^5–7^ in analyzing the experimental data on the oxidation and corrosion of metals and alloys in the wide range of the exposure time fails to find good agreement between them^8–12^.

Although the parabolic rate constant (PRC) has been claimed to be relevant to the high temperature oxidation of metals^13^, alloys^12^, and silicon^14^, there are some counter-examples to rule out the validity of the parabolic law in studying the oxidation of these materials. Indeed, we showed previously that the thermal oxidation of Si with a planar interface and that of a Si nanowire (SiNW) follow the time-dependent power laws^15–17^. Thus, it is incorrect to use the PRC in studying the oxidation of Si. This fact suggests that assuming the PRC is incorrect in studying the oxidation of metals and alloys as well, and further suggests that it may be inappropriate to apply the power law with a constant exponent^13,18,19^ in analyzing the experimental data on the corrosion of metals. Therefore, it is necessary to examine if the oxidation and corrosion of metals and alloys follow time-dependent power laws in the similar ways as the thermal oxidation of Si does^15–17^.

In this study, we show that the oxidation and corrosion of some of the metals and alloys indeed follow the time-dependent power laws of the similar kinds as we found in studying the thermal oxidation of Si, i.e. the exponent of the power law is not a constant but varies with the increase of the exposure period. Accordingly, it is incorrect to use the PRC in analyzing experimental data on the oxidation or corrosion of these materials^13^.

This paper is organized as follows. Two sets of the equations which represent the power-law growth of the oxide in the thermal oxidation of Si are introduced in “Two different kinds of the power laws” section. These equations are used in “Results” section to analyze experimental data on the oxidation and corrosion of some metals and alloys. Discussion on the results of our analyses and the conclusions are given in the last two sections.

## Two different kinds of the power laws

Let us first explain the equation with which the thickness of the oxide $x_{\mathrm {o}}(t)$ grown in the thermal oxidation of Si with a planar interface can be calculated^15,16^. It is given by

1 $$\begin{aligned} x_{\mathrm {o}}(t) = K \zeta (T)\left( \frac{t}{t_0}\right) ^{\nu (t)} , \end{aligned}$$

where *t* and *T* denote the oxidation time and the oxidation temperature, respectively, *K* is a function of *T* and the partial pressure *P* of the oxidant in the ambient environment^16^, whereas $t_0$ is a constant which provides the time scale for the growth of the oxide layer. Since an oxide film grows with the time scale of $10^0$ (h) in the thermal oxidation of Si, it is usually adequate to choose $t_0 = 1$ (h) in carrying out simulations of this system.

In addition, the function $\zeta (T)$ in Eq. (1) is defined by

2 $$\begin{aligned} \zeta (T) = \exp \left( -\frac{E_{D}^{eff}}{2k_BT}\right) , \end{aligned}$$

where the effective barrier ${E_{D}^{eff}}$ is given by the summation of the activation energies for the kinetic processes that the oxidant undergoes while it moves from the oxide surface to the interface, whereas $k_B$ denotes the Boltzmann’s constant.

Furthermore, the time dependence of $x_{\mathrm {o}}(t)$ is characterized by the temporal exponent ${\nu }(t)$, which is given by

3 $$\begin{aligned} \nu (t) = \frac{1}{2} + \frac{A}{1 + \sqrt{2t/{t_0}}} . \end{aligned}$$

Here, the first term corresponds to the diffusion of the oxidant, whereas the second term reflects the structural change of the substance by the reaction; $A = 0.20$ was found previously for the thermal oxidation of Si with a planar interface^15,16^. Thus, although $x_{\mathrm {o}}(t)$ is a non-linear function of *t*, the diffusion of the oxidant and the reactions of the oxidant and the material to be oxidized contribute to the exponent $\nu (t)$ in the additive manner.

The peculiar feature of Eq. (1) is that the temperature dependence and the time dependence of $x_{\mathrm {o}}(t)$ are separated from each other. This is a consequence of the fact that the time scale for the growth of the oxide thickness, $t_0 = 1$ (h), is several orders of magnitude larger than that of atomic and molecular processes involved in the thermal oxidation of Si.

For the oxidation of SiNWs, two modifications were found to be necessary in calculating the growth of the oxide thickness^17^. Between them, the first one is about the constant term in the temporal exponent $\nu (t)$ in Eq. (3). Since the oxidation of a SiNW is reaction-limited^17^, and does not become diffusion-limited even in the large *t* limit, the first term in Eq. (3) should be neglected. Hence, the temporal exponent should be changed to

4 $$\begin{aligned} \nu _R(t) = \frac{A}{1 + \sqrt{2t/{t_0}}}, \end{aligned}$$

where the coefficient *A* was found to depend on the initial radius of a SiNW^17^.

The second modification concerns the time scale ${t_0}$ on the growth of the oxide thickness. Actually, by carrying out simulations with the use of the reaction-diffusion equation, we found that ${t_0}$ depends on *T* as $t_0 = {\kappa }{\exp (-{E_{R}}/{k_B T})}$, where ${\kappa }$ and ${E_{R}}$ are positive constants^17^. Since ${t_0}$ provides the time period between the successive advancement of the interface, this counterintuitive relationship between ${t_0}$ and *T* shows that the structural relaxation in the oxide layer, which is necessary for the subsequent advancement of the interface to take place, extends from the transition region to the oxide layer when *T* is high, whereas it is restricted to the vicinity of the transition region when *T* is low.

Since the use of the temporal exponent in Eq. (4) makes $x_{\mathrm {o}}(t)$ in Eq. (1) not a monotonically increasing function of *t*, the oxide thickness is calculated by the updating procedure

5 $$\begin{aligned} {w}(t + \Delta {t}) = \max (x_{\mathrm {o}}(t + \Delta {t}) , {w}(t)), \end{aligned}$$

where the time variable is incremented by the addition of $\Delta {t}$ to *t* with $\Delta {t} > 0$ and the initial condition of ${w}(0) = 0$ is adopted. In addition, $\max (a,b)$ is a function which is defined such that it gives *a* if $a \ge b$ and does *b* otherwise. The mathematical expression of the oxidation rate according to Eqs. (1)–(4) is given in Eq. (A1) in “Suppl Appendix 1”.

In this work, we will show that the above equations can be used to study the oxidation and corrosion of metals and alloys by adjusting the coefficient *A* in Eq. (3) or Eq. (4) to each system based on the assumption that *A* is a function of the difference in the atomic structures as well as the chemical compositions between the solid-state reactant and the reaction product. Thus, the coefficient *A* is different from the Pilling-Bedworth ratio (PBR)^2,3^.

In applying the above equations to the corrosion of metals and alloys, we tried to fit the simulation results to the experimental data, and found it appropriate to change the constant $t_0$ from $t_0 = 1$ (h), which is adequate in studying the evolution of the oxide thickness in the thermal oxidation of Si, to $t_0 = 1$ (y) in studying the corrosion of metals at the atmospheric temperature.

Since the experimental data on the corrosion of metals and alloys are usually yielded at one temperature, we will denote the product of *K* and $\zeta (T)$ in Eq. (1) as $B = K \zeta (T)$ and evaluate *B* by fitting the calculation results using Eq. (1) to the available experimental data. Specifically, the coefficients *B* and *A* are determined by numerically minimizing the summation of the squares of the differences between the calculation results and the experimental data in each case.

## Results

### ’Uniform’ corrosion of copper

**Figure 1:**
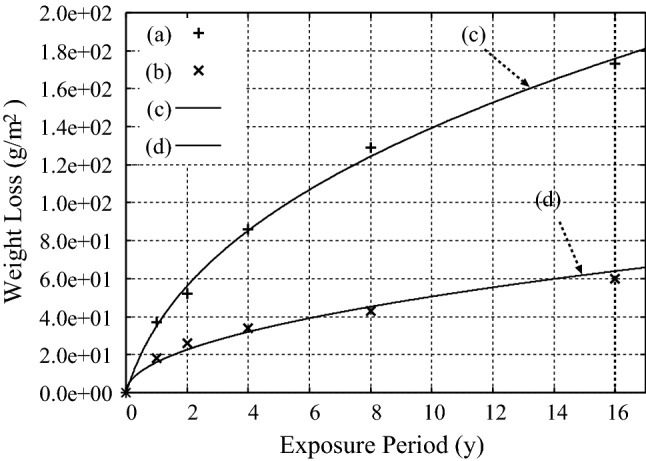
Corrosion of copper (99.9$\%$ wt.) subject to (**a**) the marine atmosphere of PCZ at Cristobal, and (**b**) the inland atmosphere of PCZ at Miraflores after Southwell et al.^20^. Results of the calculations by using Eqs. (1) and (3) for simulating the corrosion of copper subject to the marine and inland atmospheres are shown in (**c**) with $B= 3.5{\times }10^{-3}$ (mm) and $A = 0.547$, and (**d**) with $B= 1.6{\times }10^{-3}$ (mm) and $A = 0$, respectively.

The first example is the ’uniform’ corrosion of copper, for which the experimental data on the atmospheric corrosion of copper subject to the marine atmosphere of Panama Canal Zone (PCZ) at Cristobal and the inland atmosphere of PCZ at Miraflores after Southwell et al.^20^ are used, as seen in Fig. 1a,b, respectively. The calculation results are well fitted to the observation results with the use of Eqs. (1) and (3), to obtain $B= 3.5{\times }10^{-3}$ (mm) and $A = 0.547$ for the marine atmosphere and $B= 1.6{\times }10^{-3}$ (mm) and $A = 0$ for the inland atmosphere of PCZ, as seen in Fig. 1c,d, respectively.

The fact that $A = 0$ is obtained by analyzing the observed data for the corrosion of copper in the inland atmosphere of PCZ shows that the volume expansion is not associated with the chemical reaction of this corrosion process. This is realized in an aqueous corrosion, in which an incident ion diffuses and reacts with a copper ion in an aqueous electrolyte which covers the surface of the specimen. This interpretation is consistent with the results of the experiment performed by Watanabe et al.^21^.

### High temperature oxidation of stainless steel

**Figure 2:**
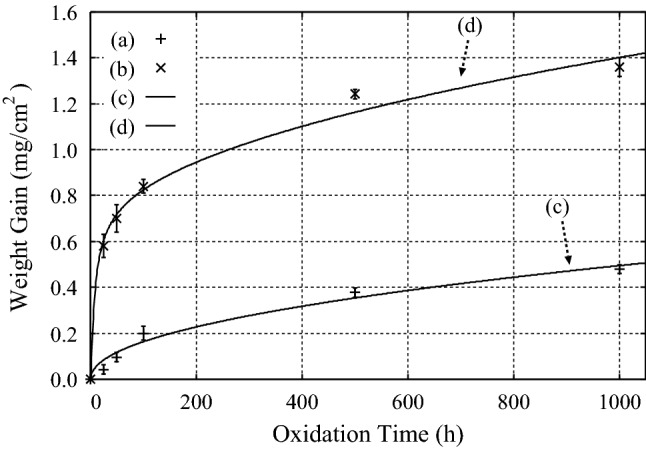
Experimental results on the weight gain of the oxidation of 316LSS (**a**) at $T = 600\,^{\circ }$C and (**b**) at $T = 800\,^{\circ }$C after Huang et al.^22^. (**c**), (**d**) Results of the calculations by using Eqs. (1) and (3) with the use of (**c**) $B= 1.50{\times }10^{-2}$ (mg/cm$^2$) and $A = 0.28$ at $T = 600\,^{\circ }$C, and (**d**) $B= 2.40{\times }10^{-2}$ (mg/cm$^2$) and $A = 4.06$ at $T = 800\,^{\circ }$C, respectively.

The second example is the high temperature oxidation of austenitic stainless steel 316L (316LSS), for which the experimental results on the weight gain by the oxidation at $T = 600\,^{\circ }$C and $800\,^{\circ }$C were reported by Huang et al.^22^. For clarity, the chemical compositions of 316LSS are listed in Table 1 in “Suppl Appendix 2”. By fitting Eqs. (1) and (3) to the experimental data, we obtained $B= 1.50{\times }10^{-2}$ (mg/cm$^2$) and $A = 0.28$ at $T = 600\,^{\circ }$C, and $B= 2.40{\times }10^{-2}$ (mg/cm$^2$) and $A = 4.06$ at $T = 800\,^{\circ }$C, as seen in Fig. 2.

The ratio of the coefficients *B* at two different temperatures is 1.6, which is rather small for the temperature difference of $\Delta {T} = 200\,^{\circ }$C. By contrast, the ratio of the coefficients *A* at these temperatures is 14.5. This large ratio reflects the fact that the chemical compositions of the oxide films grown at these temperatures are different from each other. Indeed, Huang et al. carried out the X-ray diffraction measurement and found a larger composition ratio of Cr$_2$O$_3$ in the oxide film grown at $T = 800\,^{\circ }$C than that at $T = 600\,^{\circ }$C when the composition ratios are measured at the oxidation time of $t = 100$ (h)^22^.

### High temperature oxidation of Ni-Cr alloys

**Figure 3:**
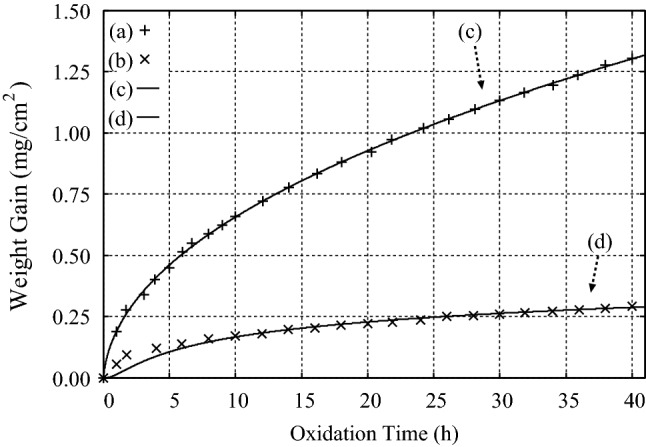
Experimental results on the oxidation of (**a**) unimplanted and (**b**) implanted (with $10^{16}$ ion/cm$^2$ Ce) Ni–30 Cr alloys at $T = 1000\,^{\circ }$C in the gas mixture of CO and CO$_2$ with the mixture ratio of 80 : 20 in mole percent, respectively, after Patibandla et al.^23^. (**c**), (**d**) Results of the calculations by using Eqs. (1) and (3) with the use of (**c**) $B= 1.92{\times }10^{-2}$ (mg/cm$^2$) and $A = 0.19$, and (**d**) $B= 1.50{\times }10^{-2}$ (mg/cm$^2$) and $A = 3.0$, respectively.

The third example is the high temperature oxidation of unimplanted and implanted (with $10^{16}$ ion/cm$^2$ Ce) Ni-30 Cr alloys at $T = 1000\,^{\circ }$C in the gas mixture of CO and CO$_2$ with the mixture ratio of 80 : 20 in mole percent, respectively^23^. By fitting Eqs. (1) and (3) to the experimental data after Patibandla et al.^23^, we obtained $B= 1.92{\times }10^{-2}$ (mg/cm$^2$) and $A = 0.19$, and $B= 1.50{\times }10^{-2}$ (mg/cm$^2$) and $A = 3.0$ for the unimplanted and implanted (with $10^{16}$ ion/cm$^2$ Ce) Ni-30 Cr alloys, respectively, as seen in Fig. 3.

These results show that both the coefficients *B* and *A* are changed by the implanted Ce atoms. In addition, the fact that $A = 0.19$, which is very close to the value of $A = 0.2$ for the thermal oxidation of Si with a planar interface, is found for the oxidation of Ni–30 Cr alloy indicates that *A* is not determined solely by the PBR^2,3^.

### Pitting corrosion of Al alloys and mild steel

The fourth example is the pitting corrosion of aluminum alloys, Al 7075, Al 2024, and Al 6061, immersed in the water of Red Sea at the temperature of $T = 21 {\pm 1}\,^{\circ }$C^24^. The chemical compositions of these alloys are listed in Table 2 in “Suppl Appendix 3”.

**Figure 4:**
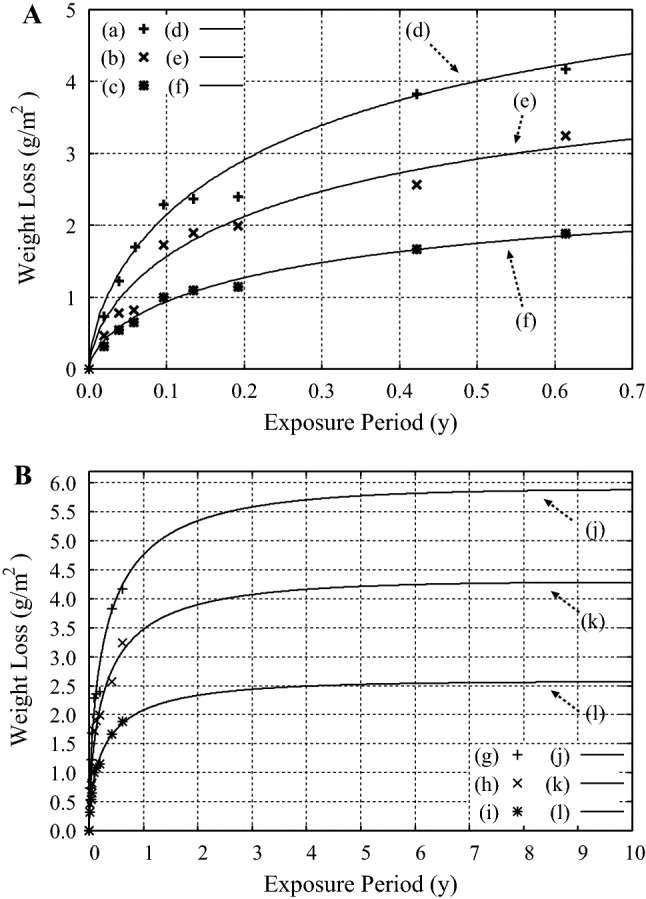
(**A**) The experimental data on the corrosion of aluminum alloys by the immersion with water of Red Sea at $T = 21 {\pm 1}\,^{\circ }$C by using the specimens of (a) Al 7075, (b) Al 2024, and (c) Al 6061 after Al-Moubaraki and Al-Rushud^24^. (d)–(f) Results of the calculations by using Eqs. (1)–(3) with $A = 0.5$ and $K = 1.0{\times }10^{7}$ (g/m$^3$) at $T = 21\,^{\circ }$C by using (d) $E_{D}^{eff} = 0.738$ (eV), (e) $E_{D}^{eff} = 0.754$ (eV), and (f) $E_{D}^{eff} = 0.780$ (eV). (**B**) Forecast of the corrosion of aluminum alloys by the immersion with seawater. (g)–(i): The experimental data at $T = 21 {\pm 1}\,^{\circ }$C for (g) Al 7075, (h) Al 2024, and (i) Al 6061 after Al-Moubaraki and Al-Rushud^24^. (j)–(l): long-term forecast of the corrosion of aluminum alloys by using Eqs. (1) and (4) plotted in the range $0 \le t \le 10$ (y) at $T = 21\,^{\circ }$C. *A*, *K*, and $E_{D}^{eff}$ are the same as those of (d)–(f).

When simulation results obtained by using Eq. (1) are compared with the experimental data after Al-Moubaraki and Al-Rushud^24^, we found good agreement between them when the exponent in Eq. (4), and not that in Eq. (3), was used in carrying out the simulations, as seen in Fig. 4A. This result shows that the pitting corrosion of these aluminum alloys is a reaction-limited process. They also show that the ’uniform’ corrosion is not realized when the aluminum alloys are immersed with seawater at $T = 21 {\pm 1}\,^{\circ }$C ^24^.

**Figure 5:**
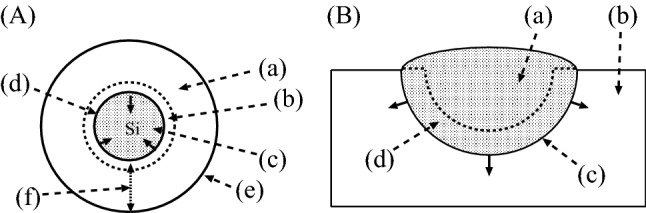
(**A**) Cross section of a SiNW in oxidation. The directions of the oxidation are indicated by the arrows in black. (a) Silicon dioxide. (b) Transition region. (c) Si core. (d) Interface. (e) Oxide surface. (f) Thickness of the oxide. (**B**) Cross section of a pit which is in growth by corrosion. The directions of the growth of the pit are indicated by the arrows in black. (a) Pit. (b) Metal or alloy to be corroded. (c) Interface. (d) Transition region.

These results can be understood by comparing them with the self-limiting oxidation of SiNWs ^25–28^, which was found to be explained well by the simulations with the use of the exponent in Eq. (4)^17^. The oxidation of a SiNW and pitting corrosion of a metal are illustrated in Fig. 5A and B, in which the directions of the oxidation and corrosion are indicated by the arrows in black.

In the oxidation of a SiNW, which is illustrated in Fig. 5A, the growth of the oxide ceases gradually because the chemical reactions in the transition region are suppressed by the stress induced by the cylindrical geometry of the oxide layer ^17,29^. Similarly, when a pit grows, large stress must be induced in the transition region, as illustrated in Fig. 5B. Therefore, the fact that good agreement was obtained between the experiments and the simulation results with the use of the exponent in Eq. (4) in this example shows that the corrosion of an aluminum alloy proceeds as a reaction-limited process by way of the pitting corrosion.

In the meantime, since the experimental data on the pitting corrosion of aluminum in Ref.^24^ were acquired at only one temperature, $T = 21 {\pm 1}\,^{\circ }$C, we could not determine the coefficient *K* in Eq. (1) or the effective diffusion energy $E_{D}^{eff}$ in Eq. (2) of the corrosive ions, which may be chloride because the samples were immersed in seawater. Therefore, the values of $E_{D}^{eff}$ are determined provisionally in this work by assuming $A = 0.5$ and $K = 1.0{\times }10^{7}$ (g/m$^3$). Accordingly, the only quantities we can derive from the results of our analyses are the changes in $E_{D}^{eff}$ according to the change in the composition of the principal diffusive ionic species. Note that $E_{D}^{eff}$ is given by the summations of the barriers for the diffusion and incorporation of the corrosive ions in the media in which the ions diffuse. See Eq. (2).

Among the chemical compositions of the alloys, the only element whose composition changes monotonically in accordance with the weight loss of the alloys by the corrosion in Fig. 4A, is Mg, as seen in Table 2 in “Suppl Appendix 3”, in which the composition of Mg in the alloys are listed as 2.1–2.9% for Al 7075, 1.2–1.8% for Al 2024, and 0.8–1.2% for Al 6061, respectively. Thus, the results in Fig. 4A indicate that the diffusion barrier of the ionic species relevant to the corrosion of the Al alloys decreases by about $2.6{\times }10^{-2}$ eV with the increase of the composition of Mg by 1 %.

Since the oxide growth in the thermal oxidation of Si with the stress-driven reaction-limited process is shown to be described by the analytic function in Eq. (1) with the combined use of Eqs. (2) and (4)^17^, the long-term behavior of the corrosion can be forecast by the simulations with the use of these equations to find that the weight loss of the Al alloys by the corrosion reaches a plateau at $t \simeq 10$ (y), as seen in Fig. 4B. In reality, however, small pits may nucleate and grow before the summation of the losses of the weights of the pre-existing pits reaches a plateau, so that the total loss of the weight may increase in the step-wise manner if the nucleation of pits takes place almost simultaneously at many different sites on the surface of a specimen.

**Figure 6:**
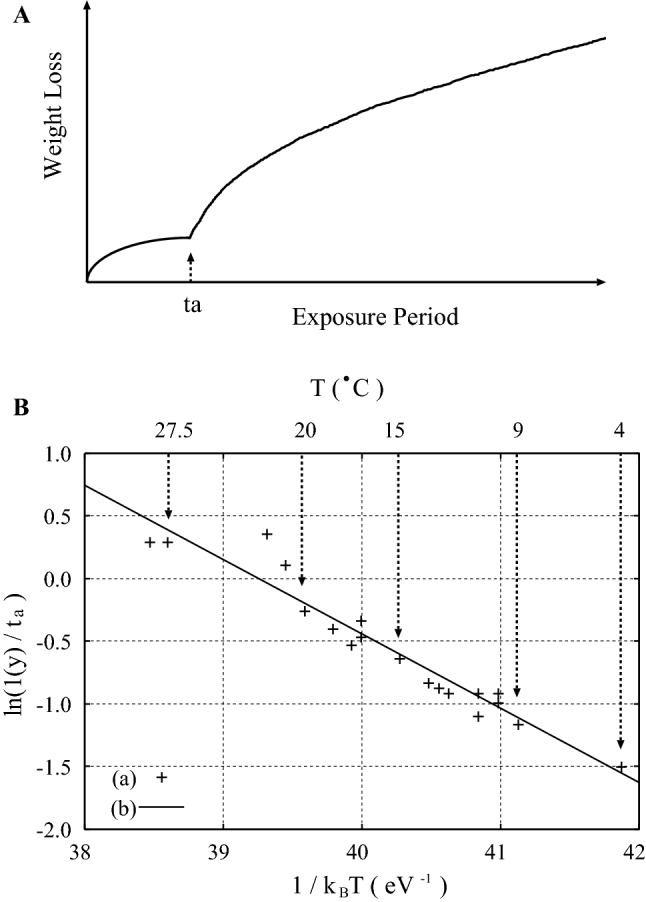
(**A**) Temporal evolution in the weight loss of the material by corrosion is schematically illustrated. The transition of the corrosion mode from the ’uniform’ corrosion to the pitting corrosion takes place at $t_a$. (**B**) Arrhenius plot of the nucleation time in the pitting corrosion of the mild steel immersed with seawater. (a) Experimental data after Melchers and Chernov^30^. (B) The line $\ln (1(\mathrm {y})/t_a) = 23.28 - E_n / k_B T$ with $E_n = 0.593$ (eV) obtained by applying the LSF to the experimental data in (A).

Although this step-wise behavior may take place for the nucleation of the pits in the first generation, it may not occur for those in the second and later generations. This is because the nucleation of pits may take place more randomly as the number of the generation increases. Here, let us denote the duration for the occurrence of the nucleation of the first generation of pits by $t_a$. Then, when the exposure period *t* exceeds $t_a$, the total loss of the weight from the body of the metal or alloy may increase almost linearly with *t*, as illustrated in Fig. 6A. By contrast, if the scale formed by the corrosion spalls off from the metal surface, the weight loss may continue to increase in the step-wise manner even when $t > t_a$.

By virtue of the nearly synchronous nucleation that many of the pits in the first generation undergo at $t \simeq t_a$, the activation energy *E*_*n*_ for the nucleation of a pit can be estimated by taking the Arrhenius plot of its inverse. By applying the least-squares fitting (LSF) to the experimental data of $t_a$ for the corrosion of the mild steel immersed with seawater after Melchers and Chernov^30^, we obtained $E_n = 0.593$ (eV), as seen in Fig. 6B, where $\ln (t_0/t_a)$ with $t_0 = 1$ (y) is plotted against the inverse temperature $1/k_B T$.

### High temperature oxidation of aluminum

**Figure 7:**
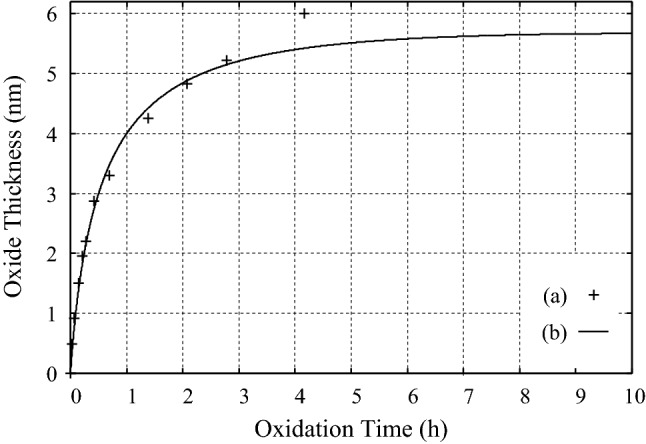
(**a**) The experimental data on the oxidation of Al(431) at $T=773$ K and $P_{\mathrm O_2} = 1.33{\times }10^{-4}$ (Pa) after Jeurgens et al.^31^. (**b**) The curve obtained by using Eq. (4) with $A=0.83$ and $B=4.09$ (nm), which are determined by applying the LSF to the experimental data in (**a**).

The experimental data on the oxidation of aluminum, Al(431), at $T=773$ K with the oxygen partial pressure of $P_{\mathrm O_2} = 1.33{\times }10^{-4}$ (Pa) after Jeurgens et al.^31^ are analyzed by using the temporal exponent in Eq. (4) to find that this process is reaction-limited with the coefficients determined as $A=0.83$ and $B=4.09$ (nm), as seen in Fig. 7.

In the experiment, oxidation was continued until $t = 1.5{\times }10^4$ (s) but the thickness of Al$_2$O$_3$ did not reach a plateau^31^. By contrast, our results show that the thickness of Al$_2$O$_3$ in the oxidation of Al(431) in the above condition reaches a plateau at $t > 10$ (h). Thus, our results show that the formation of the oxide layer on the surface of aluminum is reaction-limited and proceeds as a self-limiting process^17^.

## Discussion

In this work, we have shown that the long-term behaviors of the weight losses of metals and alloys by the corrosion and the weight gains of these materials by the oxidation can be analyzed quantitatively with the help of the two different kinds of the time-dependent power laws. The crucial role is played in the power laws by the coefficient *A* of the temporal exponent. It is worthwhile to note that the coefficient *A* depends on the structural difference between the solid-state reactant and the reaction product of the corrosion or oxidation.

Therefore, once the relationship between the coefficient *A* in Eq. (3) or Eq. (4) and the difference in the atomic structures or the chemical compositions between the solid-state reactant and the reaction product is known, the chemical composition of the reaction product can be identified by investigating the temporal change in the weight of metals and alloys in the oxidation or corrosion.

Since the equations in Eqs. (1)–(3) are the extension of the equations which were derived by analyzing the results of the simulations on the thermal oxidation of Si with the use of the reaction-diffusion equation in which the effect of the stress in the oxide, and in particular that in the transition region, is taken into account^15,16^, the physical meaning of the parameters in Eqs. (1)–(3) is known. Accordingly, the change in the diffusion barrier of corrosive ions due to the change in the chemical composition of alloys could be estimated by analyzing, e.g., the experimental data on the corrosion of aluminum alloys in Fig. 4A,B. We have also shown that the self-limiting behaviors due to the suppression of the reactions by the stress in the oxidation and corrosion of metals and alloys can be explained by using $\nu _R(t)$ in Eq. (4) instead of ${\nu }(t)$ in Eq. (3). The examples we took for showing this are the oxidation of Al alloys by the immersion with seawater at the atmospheric temperature, as seen in Fig. 4, and the high temperature oxidation of aluminum, Al(431), at $T=773$ K, as seen in Fig. 7.

Similar to these examples, the self-limiting growth of FeF$_2$ thin films reported in Ref.^32^ may also be analyzed by using Eqs. (1) and (4). By the analyses, the origin of the large difference in the limiting thickness of FeF$_2$ films found by using XeF$_2$ and SeF$_6$ may be clarified.

Here we note that, although theoretical studies found that the principal diffusive species in the oxidation of aluminum is oxygen vacancy^33^, Eq. (4) could be applied to the high temperature oxidation of aluminum, as seen in Fig. 7. This is because the oxidation takes place at the interface between the substrate and the oxide even when the principal diffusive species is oxygen vacancy.

For the purpose of investigating the atomistic processes in the oxidation and corrosion of metals and alloys, however, it is necessary to make clear the relationship between the coefficient *A* in Eq. (3) or Eq. (4) and the PBR as well as the atomic structure of the reaction product and also that between the coefficient *A* and the difference in the atomic structures between before and after the reaction.

By the comparison with various kinds of experiments, we have shown that the time-dependent power laws hold in the oxidation and corrosion of many metals and alloys. Using this property, the long-term behavior of the oxidation or corrosion of these materials can be easily forecast.

There are some more problems to be solved. One is the effect of an electric field on the diffusion of ions. Conventionally, the oxidation and corrosion of metals and alloys have been explained by taking account of the effect of the electric field between ions of adsorbed species and atoms in the surface layer of the metal substrate^5–7^. Since the field effect may change the barrier for the diffusion of ions, it may be necessary to modify the effective barrier ${E_{D}^{eff}}$ in Eq. (2) in accordance with the field applied in the diffusion or reaction of the ions.

Another problem is the temperature dependence of the corrosion process. Since metal atoms or ions diffuse into the corrosion product, the temporal evolution of the weight loss in the corrosion of an alloy may depend considerably on the temperature, and the homogeneity in the composition of the corrosion product may be lost. If such an inhomogeneity occurs, the applicability of Eqs. (1)–(4) may be lost and the extension of the equations shall be necessary in studying the temperature variation of the corrosion of alloys.

## Conclusions

The power-law ansatz found by studying the thermal oxidation of Si is used to analyze the experimental results on the corrosion and oxidation of metals and alloys. By the analyses, it is found that the weight loss of copper by atmospheric corrosion and the weight gains of austenitic stainless steel and Ni-Cr alloy by the high temperature oxidation follow the time-dependent power law in which both the diffusion and reaction processes are involved, and that the pitting corrosion of Al alloys by the immersion in seawater and the high-temperature oxidation of Al(431) follow the time-dependent power law in the reaction-limited manner. The analyses have also shown that the power law of the second kind is understood as the self-limiting process which becomes evident in the long-term behaviors of the weight changes of the reaction products such as oxide scales in the corrosion and oxidation processes. The activation energy for the nucleation of pits in the corrosion of mild steel by the immersion with seawater is estimated additionally.

## Supplementary Information

Supplementary Information.
