# Supplementary material for: Time-dependent power laws in the oxidation and corrosion of metals and alloys
Source: Sci Rep. 2022 Apr 28;12:6944. doi: 10.1038/s41598-022-10748-1 (PMC9051111; doi:10.1038/s41598-022-10748-1)
Supplement: Supplementary file 1 — Supplementary Information. [file 41598_2022_10748_MOESM1_ESM.pdf]

Appendices of Time-dependent power laws in the oxidation and corrosion of metals and alloys

Makoto Itoh[1,*]

[1]11-995 Yamanokami, Yurihonjo, Akita 015-0014 Japan
[*]mi6jpn@gmail.com

ABSTRACT

The rate of oxidation or corrosion is derived, and the chemical compositions of stainless steel 316LSS and aluminum alloys Al 7075, Al 2024, and Al 6061 are presented.

1 Rate of oxidation or corrosion

The mathematical expression of the oxidation or corrosion rate for the process in which both diffusion and reaction of the oxidant or corrosive ions are involved, respectively, is derived by differentiating $x_\mathrm{o}(t)$ in Eq.(1) with respect to t as

$$\dot{x}_\mathrm{o} = K\frac{\zeta(T)}{\tau}\left(\frac{t}{\tau}\right)^{\nu(t)-1}\left(\nu(t) - \frac{A(t/\tau)^{1/2}\log(t/\tau)}{\sqrt{2}(1+\sqrt{2t/\tau})^2}\right), \quad (A1).$$

For a reaction-limited process, the exponent $\nu_R(t)$ in Eq.(4) should be used in place of $\nu(t)$ in Eq.(3). Accordingly, the rate for corrosion or oxidation is given in terms of $\dot{x}_\mathrm{o}$ in Eq.(A1) and $\nu_R(t)$ in Eq.(4) as $\dot{w} = \max(\dot{x}_\mathrm{o}, 0)$.

2 Chemical compositions of 316LSS

The chemical composition of the austenitic stainless steel 316LSS, for which the results of the oxidation obtained by the experiments after Huang et al[1] are plotted in Fig.2(a) and (b), is listed in Table 1.

3 Chemical compositions of aluminum alloys; Al 7075, Al 2024, and Al 6061

Table 2 shows the chemical compositions of the aluminum alloys, Al 7075, Al 2024, and Al 6061, for which the results of the corrosion after Al-Moubaraki and Al-Rushud[2] are plotted in Fig.4(a)$\sim$(c), respectively.

Table 1. The chemical composition of the austenitic stainless steel 316LSS after Huang et al[1]. All elements are listed in percentages.

Alloy	Cr	Ni	Mo	Si	Mn	Cu	C	S	P
316L	16.68	12.63	2.09	0.59	1.57	0.13	0.023	0.003	0.024

Table 2. The chemical compositions of the Al alloys, Al 7075, Al 2024, and Al 6061 after Al-Moubaraki and Al-Rushudmoubaraki18. All elements are listed in percentages.

Alloy	Cr	Cu	Fe	Mg	Mn	Si	Ti	Zn	Zr	Al
Al 7075	0.18-0.28	1.2-2.0	0.5	2.1-2.9	0.3	0.4	0.2	5.1-6.1	0.25	remainder
Al 2024	0.10	3.8-4.9	0.5	1.2-1.8	0.3-0.9	0.5	0.15	0.25	-	remainder
Al 6061	0.04-0.35	0.15-0.4	0.7	0.8-0.12	0.15	0.4-0.8	0.15	0.25	-	remainder

References

1. X. Huang, K. Xiao, X. Fang, Z. Xiong, L. Wei, P. Zhu, and X. Li, Oxidation behavior of 316L austenitic stainless steel in high temperature air with long-term exposure, *Mater. Res. Express* **7**, 066517 (2020).
2. A. Al-Moubaraki and H. H. Al-Rushud, The Red Sea as a Corrosive Environment: Corrosion Rates and Corrosion Mechanism of Aluminum Alloys 7075, 2024, and 6061, *Int. J. Corros.* **2018**, 2381287 (2018).
